# Filamentous Fungi in Drinking Water, Particularly in Relation to Biofilm Formation

**DOI:** 10.3390/ijerph8020456

**Published:** 2011-02-09

**Authors:** Virgínia M. Siqueira, Helena M. B. Oliveira, Cledir Santos, R. Russell M. Paterson, Norma B. Gusmão, Nelson Lima

**Affiliations:** 1 IBB-Institute for Biotechnology and Bioengineering, Centre of Biological Engineering, University of Minho, Campus de Gualtar, 4710-057 Braga, Portugal; E-Mails: virginiamedeiros@deb.uminho.pt (V.M.S.); cledir.santos@deb.uminho.pt (C.S.); russell.paterson@deb.uminho.pt (R.R.M.P.); 2 Department of Antibiotics, Federal University of Pernambuco, Av. Prof. Morais Rego, 1235, 50670-901, Recife, Pernambuco, Brazil; E-Mails: helenambo@yahoo.com.br (H.M.B.O.); normagusmao@gmail.com (N.B.G.)

**Keywords:** fungal biofilms, drinking water, contaminants, filamentous fungi, water distribution system

## Abstract

The presence of filamentous fungi in drinking water has become an area worthy of investigation with various studies now being published. The problems associated with fungi include blockage of water pipes, organoleptic deterioration, pathogenic fungi and mycotoxins. Fungal biofilm formation is a less developed field of study. This paper updates the topic and introduces novel methods on fungal biofilm analysis, particularly from work based in Brazil. Further recommendations for standard methodology are provided.

## 1. Introduction

The issue of filamentous fungi in drinking water has matured. This is a direct result of the work of key researchers in the area [1-11], with a crucial issue being identifying isolated strains to the species, rather than simply the genus level. When species identification is undertaken more can be deduced about changes in biodiversity within locations and with time. The properties of the isolates, such as mycotoxin, pigment, and odour formation, are more reliably ascertained because these are associated more with the individual species than the genus. Furthermore, water contaminated with fungi is of importance to hospitals, where immunocompromised patients undergo treatment [12]: *Aspergillus* accounts for the majority of infections, with *Aspergillus fumigatus* accounting for 90% of cases.

Kelley *et al.* [6] provided a comprehensive investigation of filamentous fungi in US distribution systems. In this work, *Alternaria alternata*, *Aspergillus niger*, *Cladosporium* sp., *Epiccocum nigrum* and many *Penicillium* sp. were prevalent. *Penicillium brevicompactum* was rather frequently detected and *P. expansum* considerably less so. However, none of these were detected from the two tap water sites, although *Fusarium* sp. were common together with an unnamed zygomycetes. Very few *Aspergillus* sp. were detected, including *A. fumigatus*. Portuguese tap water was studied more recently [8] and Norwegian and Australian drinking water studies have provided important additional knowledge [9,10,13]. To assist the reader, the most relevant reports from water distribution systems are listed on Table 1.

## 2. Methods to Study Fungi in Tap Water

One of the most important problems for studying fungi is the use of isolation media which tends to vary among researchers. Examples of media employed are listed in Table 2. DRBC and DG18 are recommended as general media for the purpose of isolation and enumeration of fungi in foods of high water activity (aw > 0.90), and is therefore suitable for water analyses. DG18 has several advantages; the characteristic colony appearance on agar plates facilitates differentiation of species which makes secondary selection and re-isolation of pure cultures easier to accomplish, compared to DRBC. DG18 also inhibits overgrowth of fast growing fungi such as members of the Mucorales and *Trichoderma*.

Finally, DG18 does not readily produce substances toxic to the fungus when exposed to light: Media containing Rose Bengal are light sensitive and produce inhibitory compounds in significant concentrations after two hours exposure to light. The current authors recommend low and high nutritional media to obtain the complete spectrum of fungi present in water.

**Figure 1.**
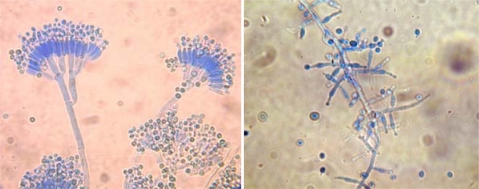
*Penicillium brevicompacum* (left) and *Trichoderma* sp. (right) isolated from tap water grown on MEA and observed under optical microscope after stained with Lactophenol Cotton Blue.

The phenotypic identification of fungi to species level is based on morphological characteristics such as (a) septation of hypha, and (b) the formation, morphology, patterns and branching frequency of fruiting-bodies and conidiophores. Also, the size, form, colour and ornamentation of the conidia are important characters (Figure 1).

**Figure 2.**
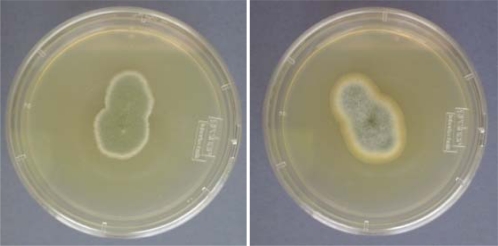
*Penicillium brevicompacum* (left) and *Penicillium aurantiogriseum* (right) isolated from tap water grown on MEA (N.B. it is significant that species names have been obtained rather than simply naming them as *Penicillium* spp.).

Macroscopic characters such as colony form, structure, size and colour are also important. In addition, appearance and growth on different culture media are used for differentiation (Figure 2). The morphological approach to species identification benefit from supplementation with physiological, biochemical and molecular characters. Ideally, a polyphasic approach to identification is undertaken to cover all aspects of species identity [20,21].

## 3. Fungal Water Biofilms

Biofilms are problematic in water distribution systems as they permit a high concentration of organisms to occur, with the potential to cause some of the problems described above. Their presence can represent a health threat since biofilms play a role in the accumulation, protection and dissemination of pathogens [11,22]. However, the involvement of filamentous fungi in biofilms has not yet been demonstrated satisfactorily due to a dearth of useful techniques.

### 3.1. Sampling Biofilms

**Figure 3.**
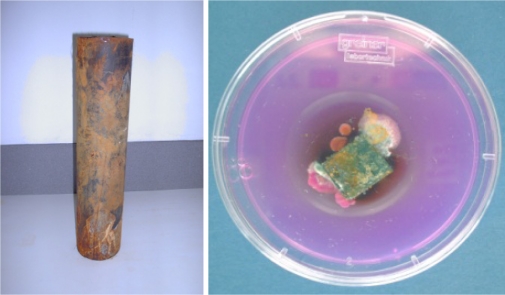
Cast iron tube from a tap water distribution system (left) and a NGRBA plate (see Table 2) with fungi growing from the biofilm present on the internal part of the tube (right).

The methods which have been employed were often indirect, where the involvement of the fungus cannot be proved categorically. An example of this is placing the inner part of pipelines in contact with fungal growth medium and allowing fungi to emerge (Figure 3).

Nevertheless, many studies on drinking water distributed systems (DWDS) have shown that the major part of the biomass is attached to pipe surfaces in a biofilm [23]. The studies of biofilms in DWDS commonly face problems such as limited access to the biofilm and difficulty in repeating experiments, since pipe sections have to be replaced after sampling. A solution is to construct sampling devices that allow for *in situ* investigation of the attached microbial community.

Examinations of DWDS reveal the complexity that will be required of such a system. Several factors are known to influence biofilm development, including temperature, nutrients, residual disinfectant, the hydraulic regime and the characteristics of the substratum [24]. Moreover, various ecological interactions will occur between microorganisms in biofilms.

Since biofilms are complex, laboratorial systems can be very helpful in determining the influence of individual parameters (e.g., medium composition and biocides application). Various attempts have been made to culture biofilms under controlled but realistic conditions, e.g., the Robbins device [25], the RotoTorque reactor [26], and flow cells [27]. However, do they reflect the *in vivo* situation?

**Figure 4.**
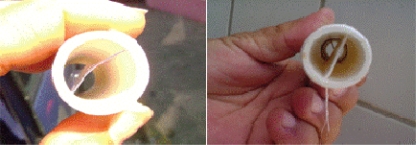
Sampler devices consisted of a hollow PVC pipe within acetate (left) or polyethylene (right) coupons.

We recommend the use of “sampler devices” to investigate natural filamentous fungi biofilms which were produced for a water distribution system called “*Alto do Céu”* in Recife, Brazil. They were developed largely because of the limitations inherent in the alternative methods mentioned previously. The devices can be employed to: (1) mimic the real conditions of the water network and yet be straight forward to insert and handle, (2) be convenient for transportation and storage, (3) maintain the integrity of biofilms and (4) allow *in situ* analyses of the biofilms. The core of the sampler devices consists of hollow polyvinyl chloride (PVC) pipes within polyethylene or acetate coupons held in place to allow biofilm growth (Figure 4).

**Figure 5.**
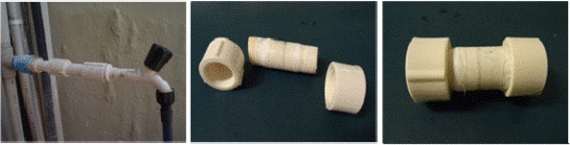
From left to right: (1) sampler device in place, (2) units of samplers to be connected to each other and (3) closed devices for transportation after a suitable period of time.

The samplers had a diameter of 1.5 cm and a length from 7 to 10 cm. The ends of each sampler form a screw to connect multiple samplers or to close the device with a cap after removal from the water network (Figure 5).

**Figure 6.**
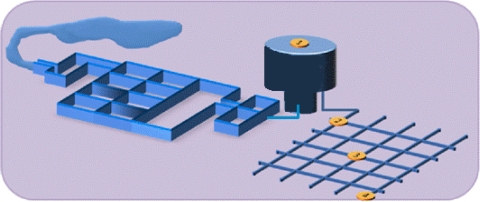
Drinking water distribution system. 1—Water treatment plant and reservoir; 2—beginning; 3—middle and 4—end of the network.

These features facilitated insertion, handling and removal of each sampler device after collection. The “caps” prevent contact with the external environment during transport. Finally, the pipes can be filled with water, thus maintaining moisture and preserving the integrity of the biofilms formed in the coupons. To study natural biofilms, these sampler devices were installed at different sampling points in the water network: a water treatment plant (reservoir; 20,000 m^3^) and three branch connections within the municipal DWDS were selected (Figure 6). The coupons were removed for examination every 15 days for a period of 6 months.

### 3.2. Biofilm Detection

**Figure 7.**
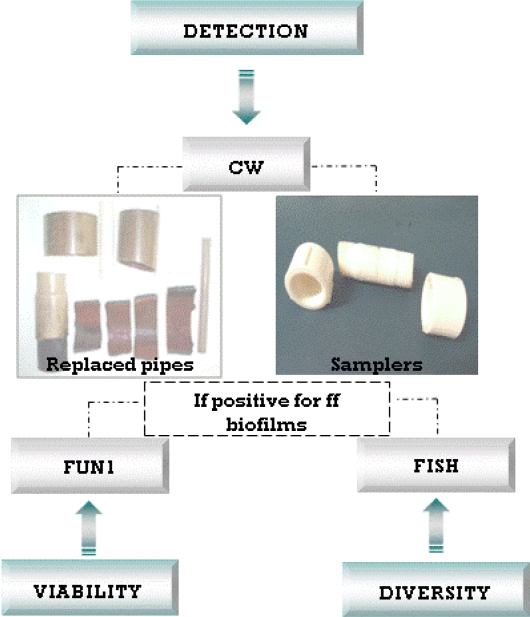
Stepwise approach used to study in situ filamentous fungal (ff) water biofilms using fluorescent staining techniques (for a description of terms see preceding paragraph).

The development of non-invasive and non-destructive techniques such as fluorescence microscopy, enables *in situ* monitoring of microbial biofilm communities. Fluorescence microscopy provides information on cell morphology, metabolism and biodiversity. Simultaneously, data concerning biofilm matrix structure and architecture are provided when used in conjunction with fluorescent molecular probes [28,29]. These data are crucial and are unattainable by conventional approaches (*i.e.*, culturing methods). They contribute to the understanding of “real time” microbial ecology within biofilms. In this context, samplers and real samples collected from *Alto do Céu* were used. The suitability of Calcofluor White MR2 (CW) staining for morphological characterisation, FUN1 for viability and Fluorescent *in situ* Hybridization (FISH) for diversity studies were investigated following the protocol presented in Figure 7.

A description of the various stains employed now follows: (1) CW is a fluorescent probe capable of making hydrogen bonds with β-(1→4) and β-(1→3) polysaccharides. CW consists of a symmetrical molecule with two triazole rings and two primary alcohol functions on both sides of an ethylene bridge. The fluorophore shows a high affinity for chitin forming hydrogen bonds with free hydroxyl groups which stains fungal cell walls blue. (2) FUN1 stain discriminates fungal dead cells which have a diffuse yellow-green fluorescence, from the metabolically active cells which have red Cylindrical Intra-Vacuolar Structures (CIVS). (3) For taxon diversity the (a) universal rRNA probe specific for Eukarya EUK516 (5′-ACCAGACTTGCCCTCC-3′, MWG Biotech, Ebersberg, Germany) labelled with the red Cy3 at the 5′ terminal and (b) FUN1429 probe specific for Eumycota (5′-GTGATGTACTCGCTGGCC-3′, MWG Biotech, Ebersberg, Germany) labelled with Oregon-Green at the 5′ terminal for FISH were employed.

**Figure 8.**
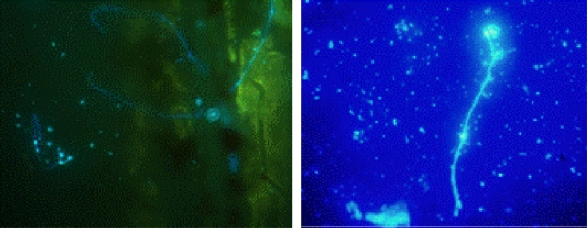
Presumptive filamentous fungal biofilm detection after CW staining on a polyethylene coupon.

A Olympus BX51 epifluorescent microscope, using UV light equipped with 40x/0.30 and 10x/0.65 objectives and a filter set (EX 450-490 nm, EM 520), was used for detecting *in situ* biofilms after fluorescent staining techniques were applied. The images were acquired with a Zeiss AxioCam HRc colour camera using the software CellB^®^. Storage and handling of reagents were performed as recommended by the supplier. The sampler study shows presumptive filamentous fungi stained with CW colonising the polyethylene coupons after 90 days in contact with water (Figure 8).

Due to the polyethylene PVC autofluorescence, the coupons inside of the samplers were replaced by acetate coupons which have much less autofluorescesce. Notwithstanding this, biofilms were not detected on acetate surfaces of coupons after CW staining.

**Figure 9.**
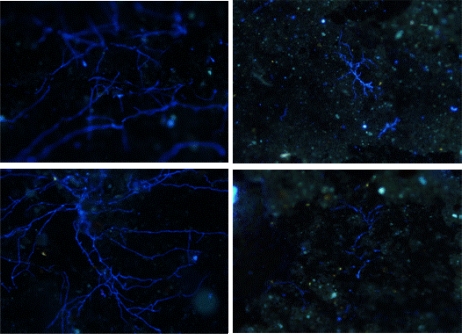
Filamentous fungal biofilms detected after CW staining in replaced pipe samples.

These unexpected results could occur for several reasons. Acetate coupons are not the optimal surfaces to promote biofilm adhesion, perhaps due to their hydrophobic properties. Alternatively, the length of time of the exposure may be inadequate for the described circumstances. These possibilities require further investigations and other materials also are required to be investigated. In contrast, when CW was applied to the actual replaced pipe samples, filamentous fungi were clearly observed (Figure 9).

**Figure 10.**
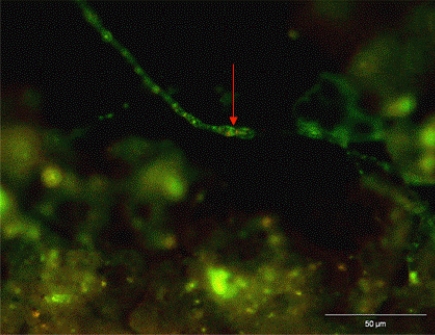
Visualization of red CIVS (arrow) and green diffuse hypha after FUN1 staining biofilm in real replaced pipe samples.

Since the real pipes demonstrated filamentous fungi within the biofilm and deposits, the stepwise approach, as defined in Figure 7, was continued. The FUN1 staining for viability showed red CIVS inside of the fungal vacuoles (Figure 10) demonstrating that the fungi were viable in the biofilm. The CIVS are ATP-dependent which is correlated with fungal viability.

**Figure 11.**
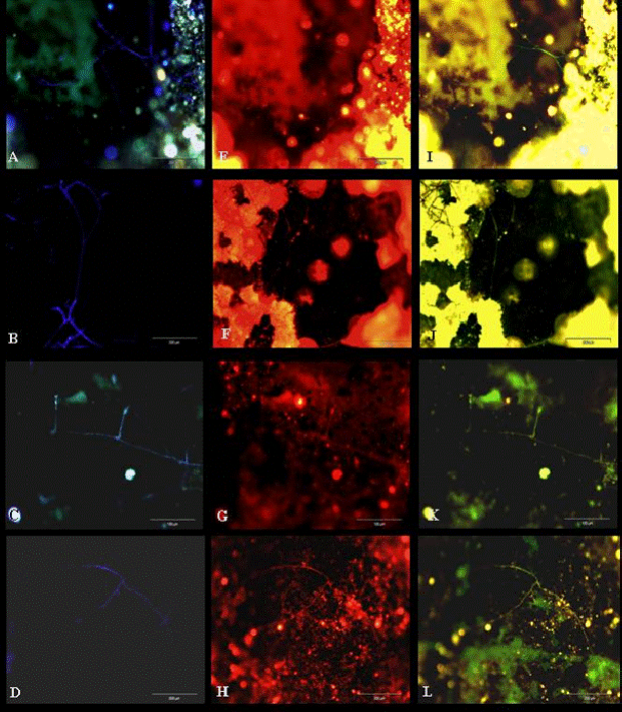
Detection of filamentous fungal water biofilm after CW staining (A-D), and analysis with FISH with EUK516 (E-H) and FUN1429 (I-L) probes in real replaced pipe samples.

To confirm that the filamentous structures in the pipes were fungi, the samples were submitted to analysis by FISH probes. Figure 11 demonstrates a clear relationship between the CW filamentous structures presented on biofilms with the positive signals for FISH probes. The EUK516 probe shows that eukaryotic microorganisms were present and the FUN1429 probe confirm that these were fungi.

## 4. Conclusions and Recommended Procedures

The present work is an updated overview concerning the main topics related to filamentous fungal contamination of water distribution system and in relation to biofilm formation. Standard methods are required, equivalent to those that exist for bacterial contamination of drinking water. The methods developed for fungal water biofilms demonstrated the presence of these microorganisms in the biofilms. Recommended standard methods for fungi have been developed and tested by the authors, and may be used to analyse water distribution systems for the occurrence of filamentous fungi [11]. The isolation method is based on NS 4716 [30] with some modifications, consist of filtering 100 mL of the water through a 0.45 μm membrane, placing the membrane on dichloran 18% glycerol agar (DG18) plates and incubating the plates at 20 ºC for 1–2 weeks. The plates must be checked visually every week, the colonies counted and the results presented as colony forming units (CFU)/100 mL water. Isolation of pure single colonies is required for identification, and pure cultures must be subcultured on potato dextrose agar (PDA) or malt extract agar (MEA) (Table 2).

To determine total filamentous fungi, the water sample must be filtered through two 0.45 μm membranes. The membranes are to be placed on neopeptone glucose rose Bengal aureomycin agar (NGRBA) with 5 replicates for filamentous fungal isolation and colony counting. The second filter should be placed onto half strength cornmeal agar (CMA/2) with five replicates for the same purposes. The method devised by us for the analysis of fungal biofilms in Brazil is also recommended more generally. Finally, even more progress will be made only if future studies ensure that strains are identified to species and standard techniques are employed universally.

## Figures and Tables

**Table 1.** Surveys of fungi in drinking water.

**Country, Place, Year**	**Period of time**	**Type of water**	**Main isolation method**	**Most frequent fungal isolates**	**Refs.**
United Kingdom, 1996	Autumn and Spring	Surface water and network	Membrane filtration, Direct plating and Bating	*Aspergillus*, *Cladosporium*, *Epicoccum*, *Penicillium* and *Trichoderma*	[4]
Greece, Thessaloniki, 1998	One collection (126 samples)	Tap water (hospital and community)	Membrane filtration	*Penicillium, Aspergillus* and *Acremonium*	[14]
Greece, 85 haemodialysis units, 1998	One collection (255 samples)	Municipal water supplies of haemodialysis centres	Membrane filtration	*Penicillium* and *Aspergillus*	[15]
Germany, North Rhine-Westphalia, 1998/9	12 months	Drinking water	Pour-plating	*Acremonium*, *Exophiala*, *Penicillium* and *Phialophora*	[5]
Norway, 14 networks, 2002/3	December, June and September	Drinking water (surface and groundwater)	Membrane filtration	*Penicillium, Trichoderma* and *Aspergillus*	[9,10]
Portugal, Braga, 2003/4	12 months	Tap water	Membrane filtration	*Penicillium* and *Acremonium*	[8]
Pakistan, Karachi, 2007	One collection (30 samples)	Water (and fruit juice)	Direct plating	*Aspergillus niger* and *A. clavatus*	[16]
Australia, Queensland, 2007/8	18 months	Municipal water	Membrane filtration	*Cladosporium*, *Penicillium*, *Aspergillus and Fusarium*	[13]
Brazil, Recife, 2009/10	5 months	Water treatment plant; tap water	Membrane filtration	*Penicillium*, *Aspergillus* and *Phoma*	[17]
Portugal, Lisbon, 2010	4 months	surface water; spring water; groundwater	Membrane filtration	*Aspergillus*, *Cladosporium*, *Penicillium*	[18,19]

**Table 2.** Principal media used for the analysis of water for fungi.

**Medium**	**Composition**	
CMA/2—Corn meal agar half-strength	Corn meal	8.5 g
	Agar	8.5 g
	Distilled water	1000 mL
	pH	5.8–6.2

CZ—Czapek Dox agar	Czapek solution	10 mL
	K_2_HPO_4_	1 g
	Saccharose	30 g
	Agar	15 g
	Distilled water	1000 mL
	pH	6.0–6.4
	Czapek solution	
	NaNO_3_	30 g
	KCl	5 g
	MgSO_4_·7H_2_O	5 g
	FeSO_4_·7H_2_O	0.1 g
	ZnSO_4_·7H_2_O	0.1 g
	CuSO_4_·5H_2_O	0.05 g
	Distilled water	100 mL

DG18—Dichloran 18 % glycerol agar	Peptone	5 g
	Glucose	10 g
	K_2_HPO_4_	1 g
	MgSO_4_·7H_2_O	0.5 g
	Dichloran (0.2% in ethanol)	1 mL
	Glycerol	220 g
	Chloramphenicol	0.1 g
	Agar	15 g
	Distilled water	1000 mL
	pH	5.4–5.8

DRBC—Dichloran Rose Bengal chloramphenicol agar	Peptone	5 g
	Glucose	10 g
	K_2_HPO_4_	1 g
	MgSO_4_·7H_2_O	0.5 g
	Dichloran	0.002 g
	Rose Bengal	0.025 g
	Chloramphenicol	0.1 g
	Agar	15 g
	Distilled water	1000 mL
	pH	5.4–5.8

MEA—Malt extract agar	Malt extract	20 g
	Peptone	1 g
	Glucose	20 g
	Agar	20 g
	Distilled water	1000 mL
	pH	5.0–5.5

NGRBA—Neopeptone glucose Rose Bengal aureomycin	Neopeptone	5 g
	Glucose	10 g
	0.67 % (w/v) aureomycin solution	5 mL
	1 % (w/v) Rose Bengal solution	3.5 mL
	Agar	15 g
	Distilled water	1000 mL
	pH	6.3–6.7

PDA—Potato dextrose agar	Potato extract	4 g
	Glucose	20 g
	Agar	15 g
	Distilled water	1000 mL
	pH	5.4–5.8

SDA—Sabouraud dextrose agar	Mycological peptone	10 g
	Glucose	40 g
	Agar	15 g
	Distilled water	1000 mL
	pH	5.4–5.8

## References

[1] Nagy LA, Olson BH (1982). The occurrence of filamentous fungi in drinking water distribution systems. Can. J. Microbiol.

[2] Nagy LA, Olson BH (1985). Occurrence and significance of bacteria, fungi and yeasts associated with distribution pipe surfaces.

[3] Franková E, Horecka M (1995). Filamentous soil fungi and unidentified bacteria in drinking water from wells and water mains near Bratislava. Microbiol. Res.

[4] Kinsey GC, Paterson RR, Kelley J (1999). Methods for the determination of filamentous fungi in treated and untreated waters. J. Appl. Microbiol. Symp. Suppl.

[5] Göttlich E, van der Lubbe W, Lange B, Fiedler S, Melchert I, Reifenrath M, Flemming H-C, Hoog S (2002). Fungal flora in groundwater-derived public drinking water. Int. J. Hyg. Environ. Health.

[6] Kelley J, Kinsey G, Paterson R, Brayford D, Pitchers R, Rossmore H (2003). Identification and Control of Fungi in Distribution Systems.

[7] Paterson RRM, Lima N, Lehr J, Keeley J, Lehr J, Kingery TB (2005). Fungal Contamination of Drinking Water. Water Encyclopedia.

[8] Gonçalves AB, Paterson RRM, Lima N (2006). Survey and significance of filamentous fungi from tap water. Int. J. Hyg. Environ. Health.

[9] Hageskal G, Knutsen AK, Gaustad P, de Hoog GS, Skaar I (2006). Diversity and significance of mold species in Norwegian drinking water. Appl. Environ. Microbiol.

[10] Hageskal G, Gaustad P, Heier BT, Skaar I (2007). Occurrence of moulds in drinking water. J. Appl. Microbiol.

[11] Hageskal G, Lima N, Skaar I (2009). The study of fungi in drinking water. Mycol. Res.

[12] Paterson RRM, Hageskal G, Skaar I, Lima N, De Costa P, Bezerra P (2009). Occurrence, problems, analysis and removal of filamentous fungi in drinking water. Fungicides: Chemistry, Environmental Impact and Health Effects.

[13] Sammon NB, Harrower KM, Fabbro LD, Reed RH (2010). Incidence and distribution of microfungi in a treated 9 municipal water supply system in sub-tropical Australia. Int. J. Environ. Res. Public Health.

[14] Arvanitidou M, Kanellou K, Constantinides TC, Katsouyannopoulos V (1999). The occurrence of fungi in hospital and community potable waters. Lett. Appl. Microbiol.

[15] Arvanitidou M, Spaia S, Velegraki A, Pezarloglou M, Kanetidis D, Pangidis P, Askepidis N, Katsinas Ch, Vayonas G, Katsouyannopoulos V (2000). High level of recovery of fungi from water and dialysate in haemodialysis units. J. Hosp. Infect.

[16] Nazim S, Dawar S, Tariq M, Zaki MJ (2008). Quantitative estimation of mycoflora in drinking water and fruit juices of Karachi. Pakistan J. Bot.

[17] Oliveira HMB (2010). Fungos filamentosos na água e em biofilmes na rede de distribuição de água potável do sistema Alto do Céu, Recife-PE.

[18] Pereira VJ, Basílio MC, Fernandes D, Domingues M, Paiva JM, Benoliel MJ, Crespo MT, San Romão MV (2009). Occurrence of filamentous fungi and yeasts in three different drinking water sources. Water Res.

[19] Pereira VJ, Fernandes D, Carvalho G, Benoliel MJ, San Romão MV, Barreto Crespo MT (2010). Assessment of the presence and dynamics of fungi in drinking water sources using cultural and molecular methods. Water Res.

[20] Santos C, Paterson RRM, Venâncio A, Lima N (2010). Filamentous fungal characterisations by matrix-assisted laser desorption/ionisation time of flight mass spectrometry. J. Appl. Microbiol.

[21] Santos C, Fraga ME, Kozakiewicz Z, Lima N (2010). Fourier transform infrared as a powerful technique for the identification and characterisation of filamentous fungi and yeasts. Res. Microbiol.

[22] Huq A, Whitehouse CA, Grim CJ, Alam M, Colwell RR (2008). Biofilms in water, its role and impact in human disease transmission. Curr. Opin. Biotechnol.

[23] Boe-Hansen R, Martiny AC, Arvin E, Albrechtsen H-J (2003). Monitoring biofilm formation and activity in drinking water distribution networks under oligotrophic conditions. Water Sci. Technol.

[24] Niquette P, Servais P, Savoir R (2000). Impacts of pipe materials on densities of fixed bacterial biomass in a drinking water distribution system. Water Res.

[25] McCoy WF, Bryers JD, Robbins J, Costerton JW (1981). Observations of fouling biofilm formation. Can. J. Microbiol.

[26] Van der Wende E, Characklis WG, Smith DB (1989). Biofilms and bacterial drinking water quality. Water Res.

[27] Caldwell DE, Lawrence JR, Wimpenny JWT (1988). Study of attached cells in continues-flow slide culture. CRC Handbook of Laboratory Systems for Microbial Ecology Research.

[28] Li Y, Dick WA, Tuovinen OH (2004). Fluorescence microscopy for visualization of soil microorganisms—a review. Biol. Fert. Soils.

[29] Gonçalves AB, Santos IM, Paterson RRM, Lima N (2006). FISH and Calcofluor staining techniques to detect *in situ* filamentous fungal biofilms in water. Revista Iberoamericana de Micologia.

[30] (1991). Determination with the membrane filters method, NS 4716. Norwegian Standard for Water Analysis: Microfungi in Water.

